# Rest for Dentists

**Published:** 1887-06

**Authors:** 


### REST FOR DENTISTS.

There is an affection peculiar to dentists which has for some time been recognized in England under the name of "Dentist's Leg." The *London Lancet* has specially called attention to it, and published more than one article upon its etiology and treatment. The symptoms are a special tingling and sensation of numbness in the leg, or what is commonly called the "pins and needles" feeling. The nutrition of the limb is finally interfered with, and it is a constant source of irritation and annoyance. Primarily, the condition is caused by the undue strain of constantly standing upon the leg in an unnatural position, and by over use in driving the dental engine. There is an impediment to natural circulation in the constant and unrelieved strain upon the muscles, and this interferes with the nutrition of the tissues. The nervous exhaustion of this continuously maintained, unnatural and strained position, is also a factor in the production of the disease.

Attention has not been called to this disorder in America as it has in England, but it is not unknown here, and many dentists have suffered to a greater or less extent without directly recognizing the source of their illness. American operators engaged in full practice not infrequently stand at the chair from eight to ten hours a day, almost continuously maintaining a constrained position, and with their mental faculties incessantly excited to the utmost. Is it any wonder that under these severe labors, as a rule, they physically fail at an early age? Their labor, too, is entirely within doors, and their habits are sedentary. When the day's toil is ended, they are too much exhausted to take the out-of-door exercise that is essential to health. Their days of rest are few, for it is too often the case that the holiday of others only brings to the dentist an increase of work, through the necessity of operating for those who can visit him at no other time. There is a large class whose time during the hours of light on every laboring day is wholly occupied, and the visits to the dentist of bookkeepers, bank clerks and many holding responsible positions, must perforce be made upon Sundays and holidays, thus depriving the dentist of needed rest and exercise.

What is he to do? He cannot wholly evade these demands upon his time, and he cannot keep his own physical system in condition without rest and exercise in the open air. It is a problem which is troubling many an anxious dentist who has, perhaps, like the writer of this, found that even a physical organization that has for many years been fully able to meet every unreasonable demand upon it, will at last find its limit and utter its protest through unaccustomed aches and pains, and even by a serious threatening of the entire stoppage of some of the most important functions of life. When this crisis arrives the operator finds it imperative that he should pull up and begin to favor himself. But where shall he commence?

One of the most exhaustive of his labors is that of standing upon one foot and running the dental engine with the other. He may employ an assistant to do this, but unless his experience differs from that of ours, he will constantly find himself back at the wheel, through the temporary absence of his assistant, the desire for special delicacy in a difficult case, or through his own nervous impatience, and thus the bounds he had set for himself are constantly overstepped. Patients, too, do not like a third person around the chair when his or her services can be dispensed with. Yet entire relief from this modern wheel of Ixion must in some way be obtained.

The crying need among dental operators is for a motor which shall be sufficient for his needs. It has not yet been found. Electricity does not answer, for this usually implies the keeping in order of a powerful battery, and any one who has tried this knows what a provoking task it is. Water motors are in many cases impracticable, because offices are frequently on upper floors, where the pressure in such places as have a water supply is too often insufficient. A miniature steam engine requires too much watching, and is dangerous when neglected. Gas and hot-air engines have not proved practicable. There is a field here for the exercise of the ingenuity of some of our inventive geniuses. A full reward awaits the man who solves the problem.

For some time we have been using a water motor that fully answers our purpose. But we have a pressure which can be relied upon. It is not heavy—about fifteen pounds to the inch—but it is steady. Immediately beneath the floor of the operating room a small sized Tuerck motor is placed, with supply and waste water pipes, and in the former a valve, which is easily controlled by the foot. The driving cord of a suspension engine passes through the floor and around the pulley of the motor, instead of about the wheel connected with a foot-treadle. That is all there is to it—simply the substitution of the motor for the usual driving wheel. It is always ready, there is nothing to clean or keep in order, and the power is abundant. For those who have a constant water supply, it is all that need be wished. It can be set upon the floor beside the chair, and a hat will cover it, and there need be no leakage or dirt from it to soil the carpet. In summer it will also drive a fan or cooling apparatus. It uses but little water, and the cost of the apparatus is small. But it is useless without a steady water supply.

There is another thing which the operative dentist will find a great convenience and relief, and that is an operating stool. If he will acquire the habit of sitting at his work, it will relieve the constant strain upon the legs. Indeed, it is this which *The Lancet* recommends as the cure for what it denominates "Dentist's Leg."

The overstrained muscles must have rest, and this can only be secured by a sitting posture while at work. American dental chairs have reached such a pitch of perfection that it is comparatively easy for the operator to bring his patient to any position that he may desire. It is not necessary for him to take up a constrained position over the patient, for the chair can be so arranged as to present either side, and almost any aspect of a tooth to the dentist. It requires some practice, but the dentist who has once acquired the habit of posturing his patient properly and then sitting while operating, will never again attempt to stand for hours supported by but one set of muscles.

It is entirely within the limits of practicability for the operative dentist who has anything like an established *clientèle*, to control his time. His patients soon learn what are his office hours, and conform to them. If he takes one or two hours for his lunch, he will find that it will be but a little time before his office will be deserted at that time. If he establishes the rule of a whole or half holiday upon Saturday, it will soon be widely known, and his patients will be quite as well suited, unless in extraordinary cases. If all the operative dentists of a city could be prevailed upon to close their offices upon Saturday afternoons, and spend that half day in pursuit of health and pleasure, each and every one of them at the end of the year would find himself better for it, not only physically but financially. Why should not this become universal among dentists? Some of our leading societies could not be engaged in a better work than the attempt to bring this about.

